# On the mechanism of marine fouling-prevention performance of oil-containing silicone elastomers

**DOI:** 10.1038/s41598-022-15553-4

**Published:** 2022-07-12

**Authors:** Stefan Kolle, Onyemaechi Ahanotu, Amos Meeks, Shane Stafslien, Michael Kreder, Lyndsi Vanderwal, Lucas Cohen, Grant Waltz, Chin Sing Lim, Dave Slocum, Elisa Maldonado Greene, Kelli Hunsucker, Geoffrey Swain, Dean Wendt, Serena Lay-Ming Teo, Joanna Aizenberg

**Affiliations:** 1 John A. Paulson School of Engineering and Applied Sciences, Harvard University, Cambridge, MA 02138 USA; 2 Wyss Institute for Biologically Inspired Engineering, Harvard University, Cambridge, MA 02138 USA; 3 Department of Coatings and Polymeric Materials, North Dakota State University, Fargo, ND 58102 USA; 4 University of San Diego, 5998 Alcala Park, San Diego, CA 92110 USA; 5 Center for Coastal Marine Sciences, California Polytechnic State University, San Luis Obispo, CA 93407 USA; 6 Tropical Marine Science Institute, National University of Singapore, Singapore, 119227 Singapore; 7 Stellwagen Bank National Marine Sanctuary, National Oceanic and Atmospheric Administration, Scituate, MA 02066 USA; 8 Center for Corrosion and Biofouling Control, Florida Institute of Technology, Melbourne, FL 32901 USA

**Keywords:** Soft materials, Polymers, Marine biology, Biomedical engineering

## Abstract

For many decades, silicone elastomers with oil incorporated have served as fouling-release coating for marine applications. In a comprehensive study involving a series of laboratory-based marine fouling assays and extensive global field studies of up to 2-year duration, we compare polydimethylsiloxane (PDMS) coatings of the same composition loaded with oil via two different methods. One method used a traditional, one-pot pre-cure oil addition approach (o-PDMS) and another method used a newer post-cure infusion approach (i-PDMS). The latter displays a substantial improvement in biofouling prevention performance that exceeds established commercial silicone-based fouling-release coating standards. We interpret the differences in performance between one-pot and infused PDMS by developing a mechanistic model based on the Flory–Rehner theory of swollen polymer networks. Using this model, we propose that the chemical potential of the incorporated oil is a key consideration for the design of future fouling-release coatings, as the improved performance is driven by the formation and stabilization of an anti-adhesion oil overlayer on the polymer surface.

## Introduction

The prevention and removal of marine biofouling on submerged surfaces remains a key global challenge in industries such as power generation, remote monitoring/sensing, and, most importantly, shipping. It is responsible for an estimated $60 billion in additional fuel and maintenance costs^1^. The commercial shipping industry alone produces 1 billion metric tons of CO_2_ emissions (3% of global emissions). Historically, the impact of marine fouling accumulation has even been cited as the deciding factor in the outcomes of war^2^ (e.g., Spanish-American War), further highlighting the importance of mitigating this unwanted biological buildup through the application of fouling-prevention treatments. The currentdominant copper-based ablative coating technology increasingly faces regulatory pressure due to more stringent environmental discharge rules^3^. This biocidal technology also faces reduced efficacy due to the emergence of copper-resistant fouling communities^4^. Given the challenges faced by copper-based ablative paint, novel approaches to marine fouling prevention have been of significant research interest, especially since the global ban of the highly potent tributyl tin (TBT) biocidal paint alternative in 2008^3^.

In the last three decades, fouling-release (FR) coatings, which minimize the adhesion of marine fouling organisms, have emerged as a potential alternative^3,5,6^. While many complex FR coating formulations incorporating amphiphilic properties^7^, hybrid material approaches^8^ or active substances^9^ have been developed and commercialized in recent years, most FR coatings are fundamentally based on the incorporation of low-viscosity silicone oils into a silicone elastomer. These incorporated silicone oils not only improve the FR coating performance, but have also shown to be biodegradable^10–14^ mitigating concerns about the accumulation of these compounds in the marine environment^15,16^. As shown in previous studies, the pre-cure addition of ‘free’ (unbound to the polymer matrix) silicone oil into the coating matrix increases surface hydrophobicity and slipperiness, enhancing the FR performance of the coatings against marine fouling organisms^17–21^. A general consensus of these studies was that a slight incompatibility between the silicone oil and elastomer (e.g., the use of a methyl-terminated oil in phenyl-terminated matrix) was preferable to full compatibility between the oil and matrix^21,22^.

In this study, we propose an alternative approach to the process of incorporating silicone oil into the elastomer matrix that entails adding a *fully compatible silicone oil* to the polydimethylsiloxane (PDMS) elastomer *in a simple post-cure infusion procedure*. The resulting surface, coined i-PDMS (infused PDMS), has been demonstrated to have FR and fouling-prevention properties^7,23–28^ that notably exceed the performance of silicone surfaces produced using a pre-cure addition of oil described in literature^17–19,27^. These i-PDMS surfaces have been shown to match and exceed the performance of the commercially available silicone-elastomer fouling-release coatings Intersleek 700 and Intersleek 900 in laboratory studies against marine bacteria (*Cellulophaga lytica*), several mussel species (*Geukensia demissa*, *Perna viridis*, *Mytilus edulis*), and a barnacle model (*Amphibalanus amphitrite*)^24,25^.

The i-PDMS in this study has a significantly higher silicone oil content than the commercial FR coatings (estimated × 5 higher) and its improved properties could potentially be attributed to this higher oil loading. We demonstrate however that it is the post-cure infusion process and not the elevated oil content that determines i-PDMS performance, by comparing i-PDMS with a pre-cure addition silicone coating, coined o-PDMS (one-pot PDMS), containing the same amount of silicone oil as i-PDMS (45–50 wt% of the silicone matrix). We provide a mechanistic hypothesis underpinning the exceptional performance of i-PDMS and discuss the potential limitations and implications of this alternative approach to silicone FR coating technology.

In addition to utilizing a comprehensive set of laboratory-based fouling-prevention assays, we have validated the performance of i-PDMS in a global marine fouling setting. We present data comparing the performance of i-PDMS, o-PDMS and the Intersleek 700 silicone FR coating at various field sites around the world. These field sites were chosen to test coating performance in temperate, sub-tropical and tropical climatic and ecological zones and across a wide range of geographic locations with Atlantic Ocean and Indo-Pacific tests sites being included in this study. As a previous study has shown^26^, assessing the coating performance trends across a diverse range of field sites, which harbor different fouling pressures and organisms, is an essential step to confirm the broader applicability of the findings presented. Only coating technologies that can demonstrate fouling-prevention performance across all oceanic environments have a realistic chance to be translated into commercially relevant fouling-release coating applications.

## Results

### Basic coating characterization

**Figure 1:**
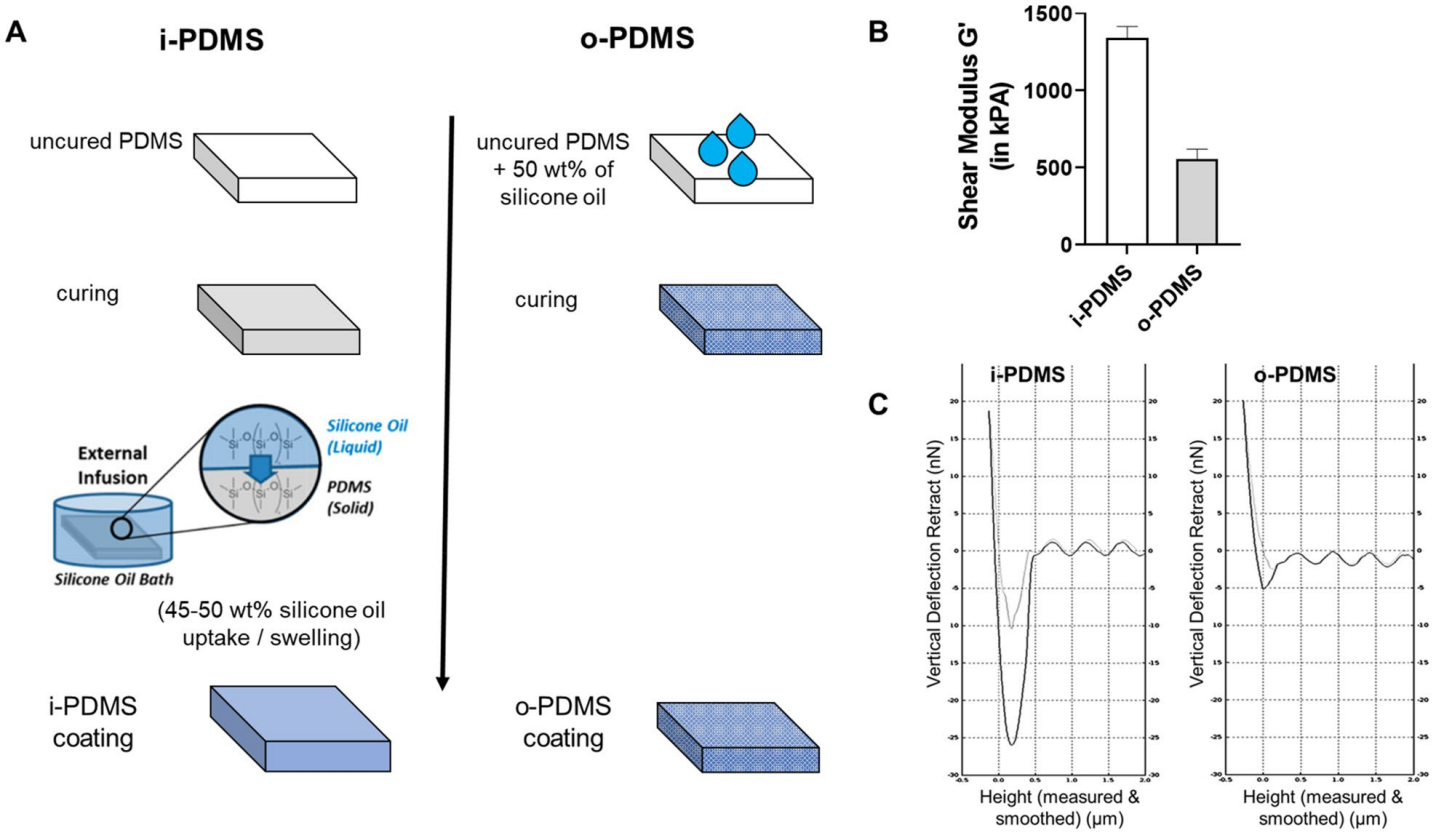
Description of the i-PDMS and o-PDMS systems. (**A**) Schematic of the fabrication processes for i-PDMS (post-cure oil infusion) and o-PDMS (pre-cure oil addition) coatings. (**B**) Shear modulus of i-PDMS (white bar) and o-PDMS (gray bar). (**C**) Lubricant overlayer detection using atomic force microscopy (AFM). The light grey curve represents the “extend” curve (the AFM tip approaching and then contacting the sample). The grey blue is the inverse “retract” curve, showing the adhesive force experienced by the AFM tip. This adhesive force is much higher for i-PDMS (~ 26 nN) compared to o-PDMS (~ 5 nN) due to the presence of the lubricant layer on i-PDMS.

Supplementary Information S1 provides details on sample preparation and analysis. The i-PDMS and o-PDMS coatings are essentially identical in composition; each contains ~ 50 wt% free silicone oil within the PDMS matrix. All observed differences in material properties arise from the fabrication process of the coatings, where the oil is added either before curing (o-PDMS) or infused into the polymerized material after curing the PDMS matrix (i-PDMS) (Fig. 1A).

The i-PDMS, o-PDMS, and oil-free PDMS control coatings were adhered to a steel or glass substrates. The coating thickness was initially set at ~ 100 µm for PDMS control and o-PDMS coatings. Due to the swelling process involved in the production of the i-PDMS from the PDMS control coating, the i-PDMS coating thickness increased to ~ 150 µm. The Intersleek 700 treatment also produced a coating with the thickness of ~ 150 µm according to manufacturer instructions. After lubricant infusion and the removal of excess lubricant (through centrifugal force or drainage), i-PDMS was measured to be 2.4 times stiffer than o-PDMS (through nano-indentation) due to the swelling of the i-PDMS coating (Fig. 1B). Moreover, the i-PDMS surface appeared coated with a lubricant overlayer (LOL) that was stable in air and resisted basic removal attempts such as exposure to running water^28^. In contrast, the o-PDMS coating did not display any lubricant on the coating surface (Fig. 1C). While the water contact angles (CA) for i-PDMS and o-PDMS were similar (113° ± 0.7° and 110.4° ± 1.1°, respectively), the coatings showed a greater difference in terms of contact angle hysteresis (CAH) (2.1° ± 0.7° for i-PDMS and 8.9° ± 2.6° for o-PDMS), indicating a higher degree of slipperiness and lower water pinning for the i-PDMS treatment.

### Laboratory evaluation of i-PDMS and o-PDMS fouling prevention performance

The biofouling performance of four distinct elastomeric anti-adhesive coatings (i-PDMS, o-PDMS, Intersleek 700 and PDMS control) were compared under laboratory conditions, following accepted Office of Naval Research (ONR) and industrial testing procedures (see Supplementary Information, S1: Method section). The testing was done with single species adhesion, attachment and retraction experiments making use of the adhesion properties of bacteria (*Cellulophaga lytica*), diatoms (*Navicula incerta*), mussels (*Geukensia demissa*) and barnacles (*Amphibalanus amphitrite*) in collaboration with North Dakota State University (NDSU).

**Figure 2:**
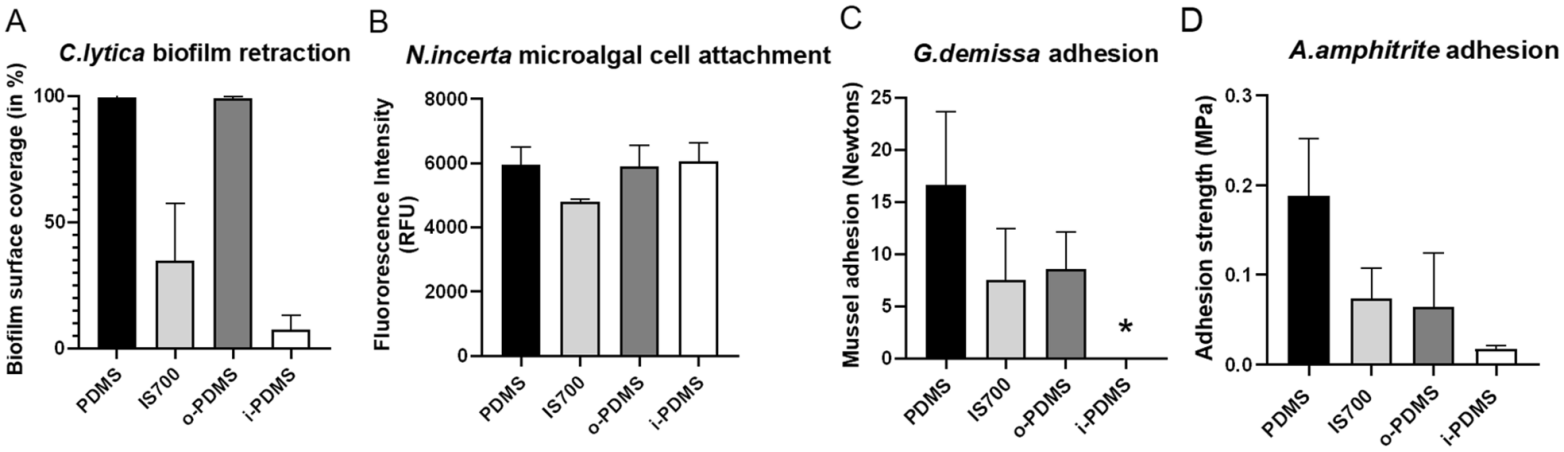
Comparative performance of o-PDMS, i-PDMS, Intersleek 700 and a PDMS control coating in marine fouling assays. Surfaces were tested for anti-adhesion performance against (**A**) bacteria (*Cellulophaga lytica*) (N = 3), (**B**) microalgal diatoms (*Navicula incerta*) (N = 4), (**C**) mussels (*Geukensia demissa*) (N = 6) and (**D**) barnacles (*Amphibalanus amphitrite*) (N = 6). Error bars = standard deviation (SD). * Signifies no adhesion took place.

As shown in Fig. 2A the i-PDMS coating had the best performance in the *C. lytica* biofilm retraction assay and had the smallest remaining biofilm coverage (7.41% ± 5.74%), followed by Intersleek 700 (34.74% ± 22.83%). PDMS control and o-PDMS showed no retraction at all in this assay. None of the coatings showed a strong performance in the *N. incerta* microalgal assay, with only Intersleek 700 showing a slightly reduced fluorescence intensity (4811 RFU ± 75 RFU), indicating lower microalgal attachment to the coating (Fig. 2B). During the *G. demissa* adhesion assay none of the mussels adhered to the i-PDMS coating, further supporting the strong performance of i-PDMS against this particular group of hard-fouling organisms (Fig. 2C)^25^. In comparison, mussels readily adhered to PDMS control, IS700 and o-PDMS during the assay. i-PDMS was also the best performing coating in the *A. amphitrite* barnacle adhesion assay, with barnacle adhesion strength to the i-PDMS (0.018 MPA ± 0.003 MPA) being significantly lower than that of PDMS control, o-PDMS and Intersleek 700 (Fig. 2D). In summary, i-PDMS showed a significantly stronger fouling-release performance than o-PDMS during all assays, with the exception of the *N. incerta* microalgal assay, where neither of the two coatings performed differently from the PDMS control. The results summary and the statistical analysis for the laboratory studies can be found in Supplementary Information S2.1 Tables 1–5.

### Field evaluation of i-PDMS and o-PDMS fouling prevention performance

#### Field immersion studies at Scituate Harbor, MA, USA (North Atlantic, temperate climate zone)

**Figure 3:**
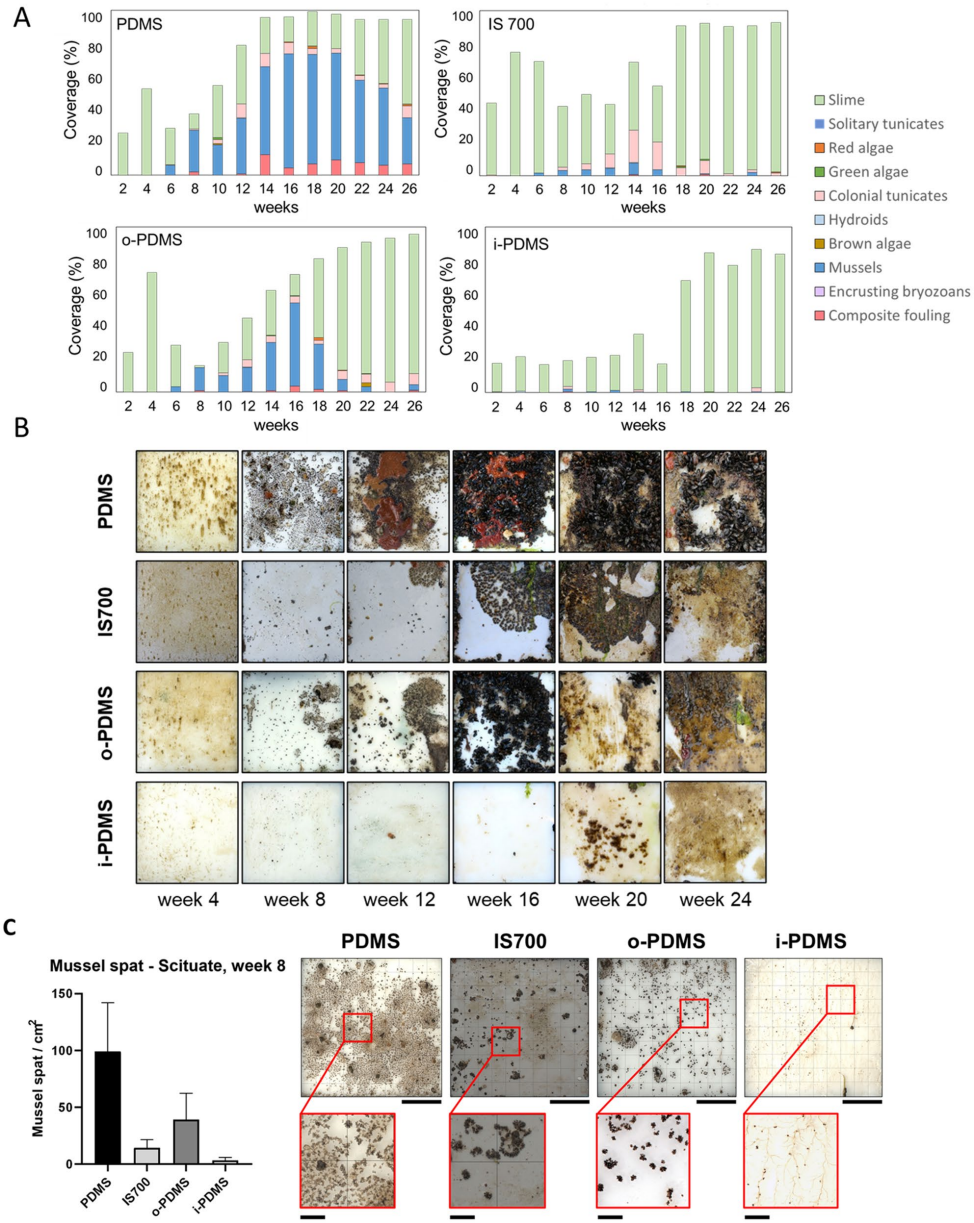
(**A**) Fouling coverage and composition on PDMS control, Intersleek 700 (IS700), o-PDMS and i-PDMS over a 6-month emersion period at Scituate Harbor, MA. Composite fouling = the combination of hard and soft fouling organisms growing on top of another, this is considered to be the heaviest, most problematic fouling category. (**B**) Representative images of the treated panels (17.5 cm × 17.5 cm) showing the fouling trends observed on each coating. Number of samples for each coating type (N) = 5. (**C**) Mussel spat densities on PDMS, IS700, o-PDMS and i-PDMS treatments in week 8. Error bars = standard deviation (SD) with presentative images on the mussel spat accumulation patterns on each treatment type. Note that on i-PDMS surface, mussel spat generally forms on remnants of the retracted biofilm, as shown in insets. Full panel scale bar = 5 cm, inset scale bar = 1 cm, number of samples (N) = 5.

The field experiments at Scituate were conducted in collaboration with Stellwagen National Marine Sanctuary (NOAA) (see Supplementary Information S1 for details on sample preparation and analysis). Regular (bi-weekly) access to Scituate field site allowed for a high temporal resolution study of the fouling community development over a 6-month period (May–November 2014) (Fig. 3A).

The first fouling community to establish was a thin biofilm (or ‘slime’) on all surfaces until week 4 (Fig. 3B). From week 6 onwards these biofilms started to recede, and mussel spat (*Mytilus edulis*) started to settle on all coatings. The settlement of the mussels was quantified at week 8, when the mussel spat was large enough to be counted (approximately 0.5 mm in length) (Fig. 3C). The aggregation patterns of the mussels differed between the coatings. On the PDMS control the mussel spat settled densely and spread out over the coating surface. On o-PDMS and Intersleek 700, the mussel spat showed a clumped distribution. Very few mussels settled on i-PDMS and their presence appeared largely limited to retracted biofilm remnants, rather than the coating surface itself (Fig. 3C). In agreement with the laboratory assays shown in Fig. 2C, the total number of mussels settled was significantly different (α ≤ 0.05) between all treatments with the lowest numbers of mussels settling on i-PDMS (3.4 ± 2.6 mussels/cm^2^), followed by Intersleek 700 (14.3 ± 7.3 mussels/cm^2^), o-PDMS (39.3 ± 23.1 mussels/cm^2^) and PDMS control (99.1 ± 46.8 mussels/cm^2^) (Fig. 3C). The results summary and the statistical analysis for the mussel spat densities can be found in Supplementary Information S2.2 Tables 6 and 7.

Over the following weeks and months, the fouling communities developed differently on each coating. The small number of mussels attached to i-PDMS disappeared completely by week 12, with most of the coating surface (65–80%) remaining free of fouling until week 18 (early October) when thicker algae biofilms started to cover most of the coatings surface. The initial 16-week delay in fouling build-up was at least partially due to strong biofilm retraction effects (similar to the ones depicted in laboratory assays shown in Fig. 2A), which removed most of the attached fouling at each sampling point. Aside from i-PDMS, no other coating showed any biofilm retraction effects.

The mussel communities attached to Intersleek 700 grew until week 16, after which most of the mussel disappeared from the coatings surface. Mussel coverage never exceeded more than 10% on Intersleek 700 during the study. The coatings surface was mostly dominated by thin biofilms during the study period, with some colonial tunicate, soft-fouling coverage (up to 20%). By the end of the study, most of the surface was covered in thick algal biofilms with no mussels and little (< 5%) colonial tunicate coverage remaining by week 26.

The mussel communities on o-PDMS grew substantially, until its coverage was > 50% by week 16. Thereafter, the increased weight of the mussels led to a series of fouling-release events, some of which took place during the field surveying of the coated panels. Mussel coverage declined drastically until week 26, when most (> 80%) of the o-PDMS surface was covered in a thick algal biofilm, with little (< 5%) mussel and low (< 10%) colonial tunicate coverage remaining.

The mussel communities on PDMS control expanded rapidly after settlement in week 6 and by week 16, > 70% of the coatings surface was covered. The mussel fouling on PDMS control followed the seasonal trend seen on the other coatings and declined towards the end of the study (weeks 22–26). Nevertheless, remaining mussel coverage was highest on PDMS control (> 35%), with substantial algae biofilm (> 50%) and little colonial tunicate coverage (< 5%) also being present by week 26.

In summary, i-PDMS showed the most promising performance at the Scituate Harbor field site, effectively preventing the build-up of the dominant mussel community and delaying the onset of fouling for a period of 4 months. While o-PDMS did not prevent the build-up of the mussel community, it showed sufficient fouling-release properties by the end of the study. In contrast, the PDMS control only showed limited fouling-release properties and retained half of its hard-fouling coverage.

#### Long-term field immersion studies at Singapore Harbor, Singapore (Indo-Pacific tropical climate zone)

The field experiments in Singapore were conducted in collaboration with the Tropical Marine Science Institute (TMSI) at the National University of Singapore (NUS) (see Supplementary Information S1 for details on sample preparation and analysis). The fouling pressure at the Singapore site resulted in clear differentiation between coatings over the 24-month test period (June 2015 – May 2017). Over this period, the development of hard-fouling communities (mostly oysters, barnacles and tubeworms), soft-fouling communities (mostly macroalgae, sponges and tunicates) and microalgal biofilms (‘slime’) on the different coatings was monitored monthly.

**Figure 4:**
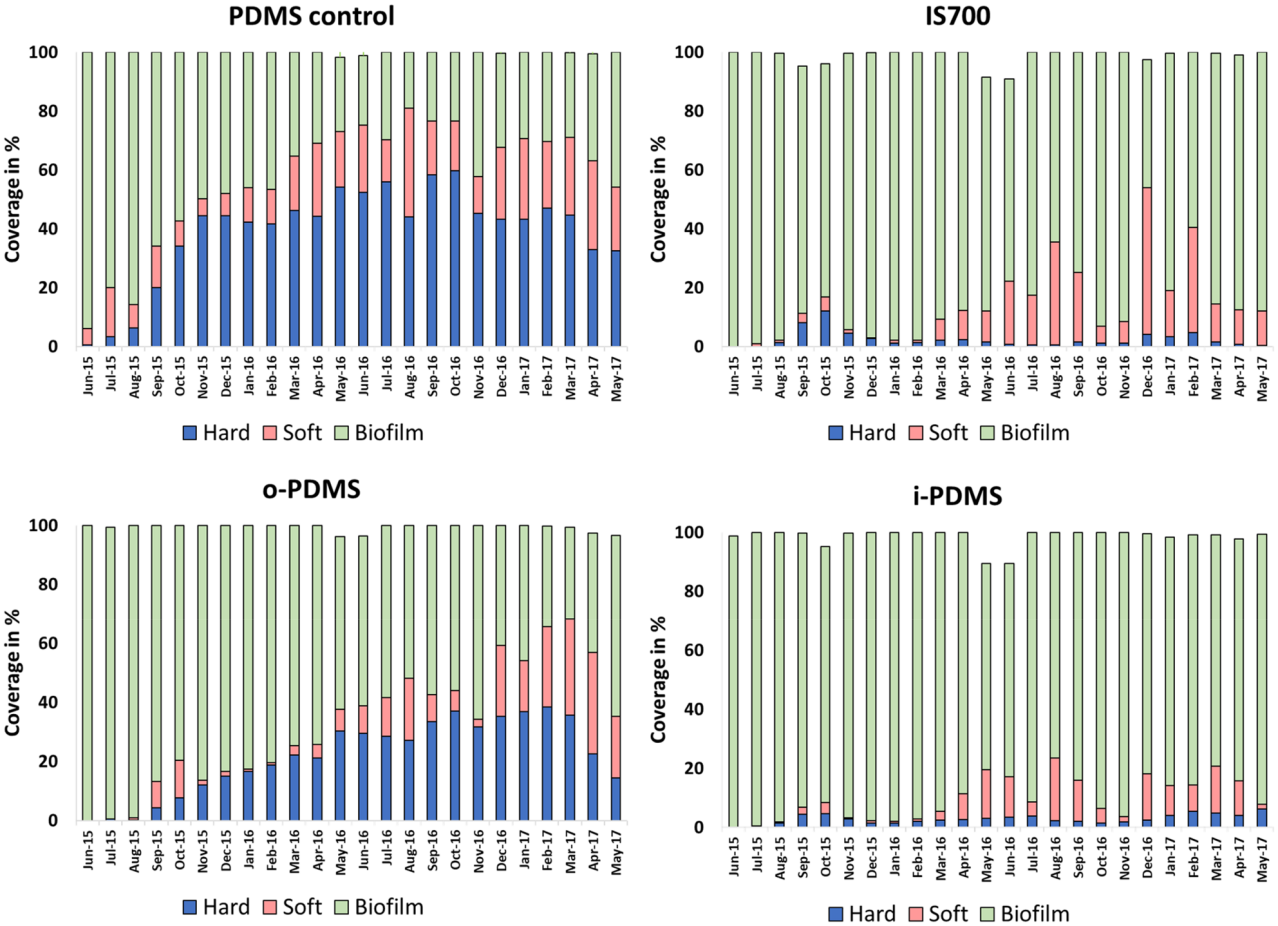
Fouling trends on PDMS, IS700, o-PDMS and i-PDMS in Singapore Harbor in a 24-month immersion period from June 2015 to May 2017. Number of samples (N) = 5.

The long-term (2 year) fouling-prevention performance of the coatings in Singapore followed similar trends to those seen in the Scituate study (Fig. 4). The i-PDMS and Intersleek 700 treatments effectively prevented the development of hard fouling communities on coating surface. Over the 24-month study period, the fouling communities on both coatings are largely dominated by microalgal biofilms, confirming the results of the NDSU microalgae assay that showed significant diatom adhesion to all coatings (Fig. 2B). Soft fouling is largely absent in the first year of the study (< 10% coverage) but becomes more prominent in the second year with a maximum soft-fouling coverage of ~ 20% on i-PDMS and ~ 45% on IS700.

The o-PDMS and the PDMS control treatments did not prevent the establishment of a hard-fouling community. A continuous hard-fouling coverage developed on these coatings 2–4 months into the study. Maximum hard-fouling coverage is > 25% for o-PDMS and > 55% for the PDMS control. In addition, both coatings develop a soft-fouling coverage with the majority of the o-PDMS and PDMS control surfaces being covered by macrofouling towards the end of the study. However, the buildup of the macrofouling community was slower for the o-PDMS (19 months until > 50% macrofouling coverage) coatings than for the PDMS control (5 months until > 50% macrofouling coverage).

In summary, the Singapore field results support the general performance trend seen in the Scituate field study and in laboratory assays, with i-PDMS showing the best fouling prevention performance, followed by IS700, o-PDMS, and the PDMS control exhibiting the least fouling prevention performance.

#### Static immersion and hard-fouling adhesion studies in Morro Bay, CA, USA (Pacific Ocean, temperate climate)

**Figure 5:**
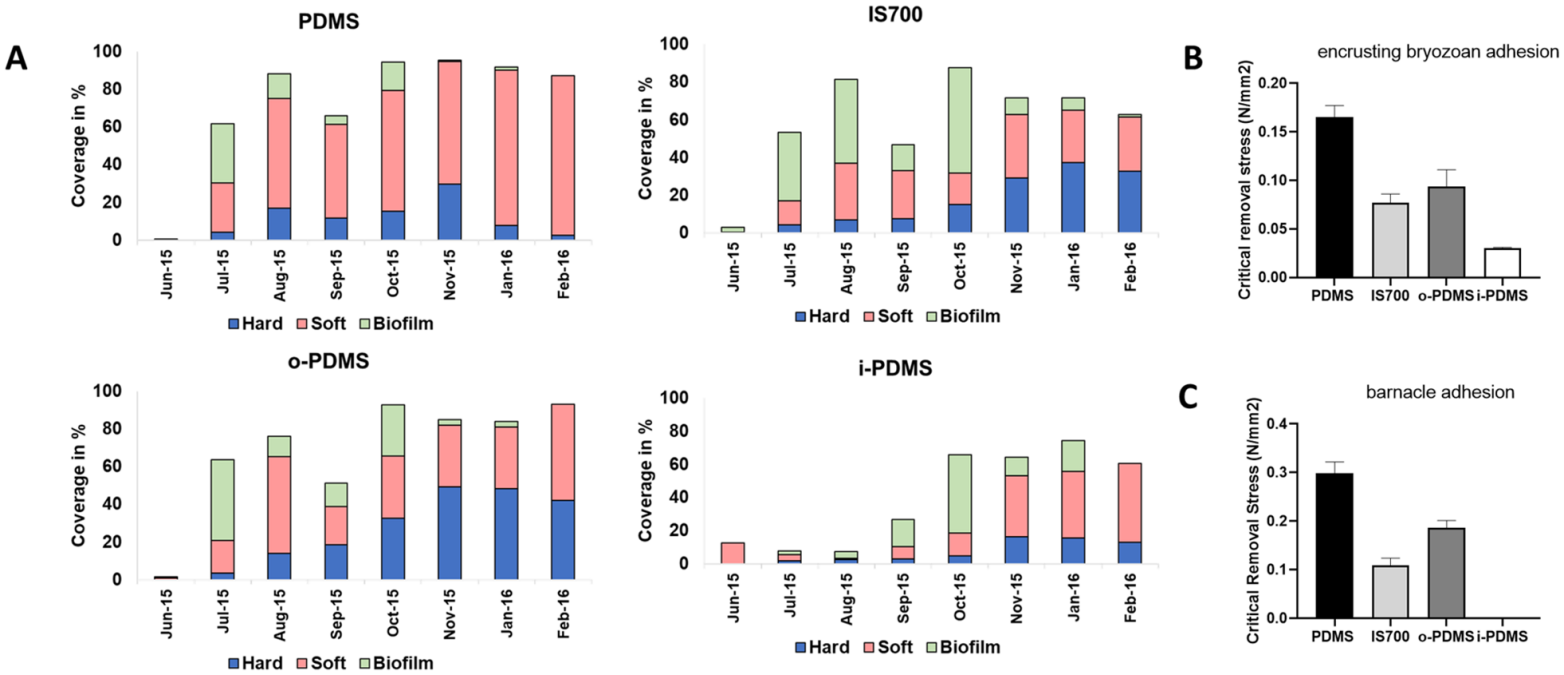
(**A**) Fouling trends on PDMS, IS700, o-PDMS and i-PDMS in Morro Bay over a 15-month immersion period from May 2015 to September 2016. The black line in each dataset corresponds with a pressure washing treatment in March 2016 removing all adhered fouling. Any growth from April 2016 onwards represents a newly established fouling community after pressure-washing the panels. Number of samples (N) = 5. (**B**,**C**) Encrusting bryozoan (**B**) and barnacle (**C**) adhesion strength to PDMS, IS700, o-PDMS and i-PDMS in Morro Bay. Error bars = standard deviation (SD).

The field experiments in Morro Bay were conducted in collaboration with the Center for Coastal Marine Sciences at the California Polytechnic State University (CalPoly) over a 15-month period from May 2015 until September 2016. In addition to the static immersion fouling studies, encrusting bryozoan adhesion and barnacle adhesion studies were conducted in July and August 2015, respectively (see Supplementary Information S1 for details on sample preparation and analysis). Over this period, both hard-fouling communities (encrusting bryozoan- and barnacle-dominated) and soft-fouling communities (colonial tunicate- and hydroid-dominated) developed on the coatings. Overall fouling coverage was lowest on the i-PDMS treatment, which maintained areas free of fouling throughout the entire study period (Fig. 5A). Interestingly, i-PDMS showed the same 4-month fouling delay previously seen in the Scituate Harbor study (Fig. 3A). Furthermore, while barnacles established themselves readily on IS700, o-PDMS and PDMS, there was little to no barnacle coverage on i-PDMS and the hard-fouling community on this coating was largely limited to encrusting bryozoans. In comparison, Intersleek 700 showed elevated hard and softfouling after one month into the study. Neither o-PDMS or PDMS control showed any particular fouling-prevention performance (Fig. 5A).

i-PDMS also performed best during the hard-fouling adhesion tests, as significantly less force was required to remove encrusting bryozoans from the coatings surface than from o-PDMS, Intersleek 700 and the PDMS control (Fig. 5B). The evaluation of barnacle adhesion strength on i-PDMS compared with the other coatings could not be conducted, as there was no barnacle settlement on i-PDMS during the study period (Fig. 5C). Barnacle adhesion was significantly lower on Intersleek 700 than on o-PDMS and significantly lower on o-PDMS than on the PDMS control. The results summary and the statistical analysis for the encrusting bryozoan and barnacle adhesion can be found in Supplementary Information S2.3 Tables 8–10.

#### Barnacle field adhesion studies at Port Canaveral, FL, USA (North Atlantic, sub-tropical climate zone)

The field experiments at Port Canaveral were conducted in collaboration with Center of Corrosion and Biofouling Control (CCBC) at the Florida Institute of Technology (FIT) (see Supplementary Information S1 for details on sample preparation and analysis). Barnacle (mostly *Balanus eburneus*) hard-fouling adhesion was tested after 4 and 7 months (in September and December 2015) of immersion, respectively. At these time points, all coatings, including a copper-based AF paint reference control, were completely covered in a thick layer of colonial tunicates which needed to be removed before any hard-fouling measurements could be attempted. The results summary and the statistical analysis for the barnacle adhesion in month 4 and 7 can be found in S2.4 Tables 11–13.

**Figure 6:**
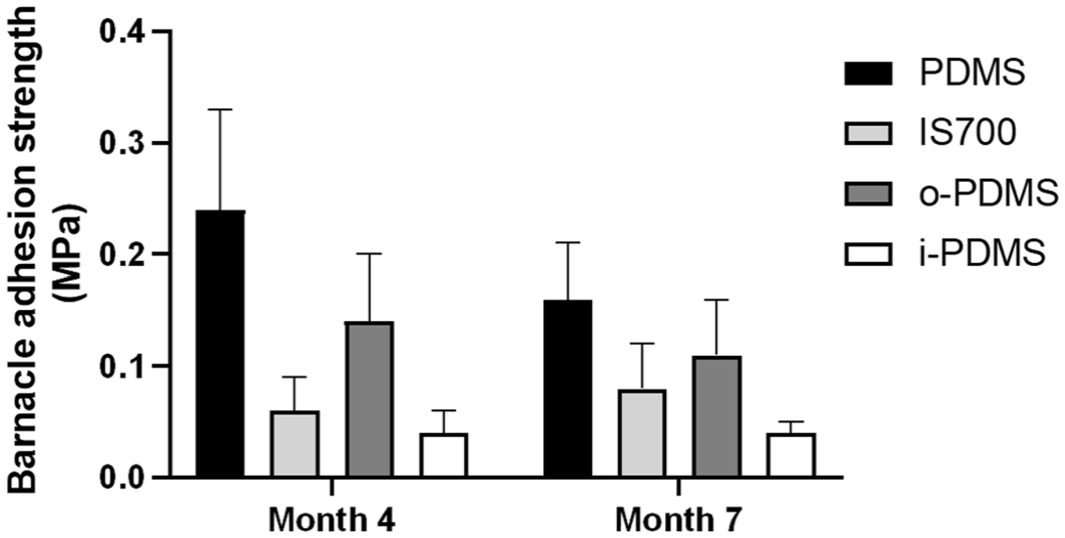
Barnacle adhesion strength to PDMS, IS700, o-PDMS and i-PDMS at Port Canaveral after 4- and 7-months static immersion, number of samples (N) = 6. Error bars = standard deviation (SD).

The subsequent adhesion measurements produced a clear trend with i-PDMS showing the lowest barnacle adhesion strength, followed by Intersleek 700, o-PDMS and PDMS control (Fig. 6). This performance trend therefore follows the same pattern as the fouling community development, mussel recruitments and hard-fouling adhesion studies conducted in Scituate Harbor, Morro Bay and laboratory assays.

### Comparing the performance of o-PDMS and i-PDMS using Flory–Rehner theory

#### Applying Flory–Rehner theory to PDMS gel swelling

**Figure 7:**
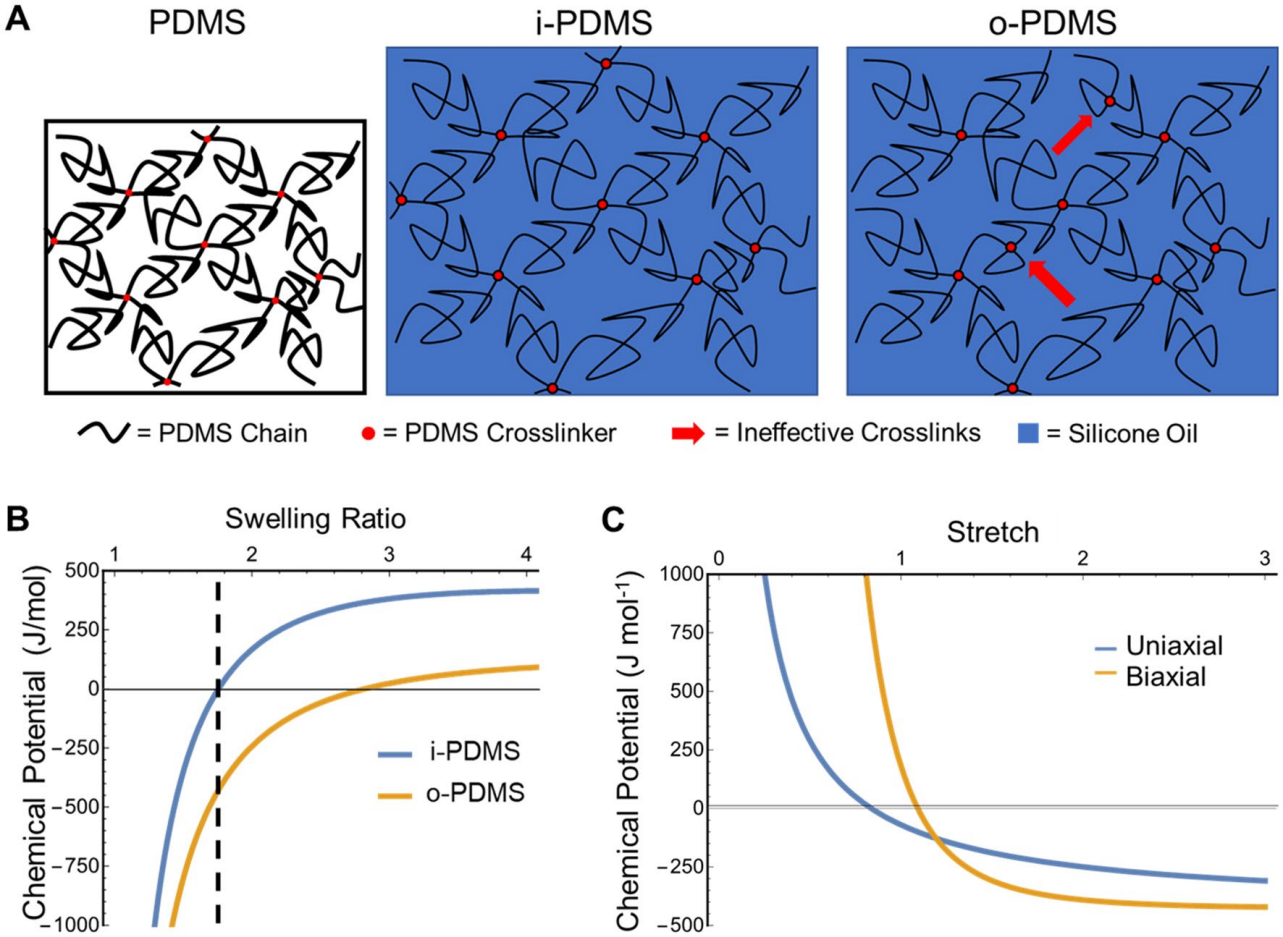
Flory–Rehner theory applied to the properties of o-PDMS and i-PDMS. (**A**) Idealized depictions of the difference in structure between i-PDMS and o-PDMS. Black lines represent the PDMS network, red dots indicate crosslinks, and the blue background represents the swelling oil. o-PDMS contains a high number of self-crosslinking, highlighted by red arrows. (**B**) Graph of the theoretical chemical potential of oil as a function of the swelling ratio. The dashed black line indicates the equilibrium swelling ratio of i-PDMS and the equivalent composition of the as-prepared o-PDMS. The o-PDMS has a large negative value for the chemical potential at this composition, indicating the much higher energy cost of removing oil from the PDMS matrix compared to swollen i-PDMS. (**C**) Chemical potential of o-PDMS as a function of uniaxial or biaxial stretch relative to the unswollen dimensions. The as-prepared stretch of 1.19 corresponds to the zero-stress state with a negative chemical potential. Compressive stress quickly raises the chemical potential, potentially removing the barrier to form a full lubricant overlayer underwater.

To explore how a small change in processing might lead to such divergent properties, we developed a mechanistic model describing o-PDMS and i-PDMS based on the Flory–Rehner theory of swelling an elastomer network in a small molecule solvent^29^. In this model, we consider two polymerization conditions, both with the same number of polymerizable monomers *m* and crosslinking molecules $\nu$, but one with some concentration of silicone oil that acts as an inert diluent in the polymer (Fig. 7A).

Flory and Rehner^29^ formulate the free energy of the swelling elastomer as being composed of a stretch term and a mixing term:

$$W = W_{stretch} + W_{mix} .$$

If we assume molecular incompressibility of an isotropically free-swelling gel, then we can write:

$$\lambda^{3} = 1 + vC,$$

where $\lambda$ is the stretch compared to the dry elastomer reference state, *C* is the nominal concentration of solvent inside of the gel (the number of solvent molecules divided by the dry state volume), and $v$ is the volume per solvent molecule. We can define the swelling ratio $J = \lambda^{3}$. When there are no applied forces on the gel, then the change in the free energy density $W$ upon swelling comes only from a change in the concentration of solvent in the gel, meaning:

$$dW = \mu dC = \frac{\mu }{v}dJ$$

where $\mu$ is the chemical potential. Rearranging slightly, we see that

$$\frac{dW}{{dJ}} = \frac{\mu }{v}$$

Flory and Rehner^29^ derived an expression for $W\left( J \right)$ for a swollen network using the Gaussian chain model and the Flory–Huggins theory of mixing:

$$W\left( J \right) = \frac{3}{2}NRT\left( {J^{\frac{2}{3}} - 1 - \frac{2}{3}\log J} \right) + \frac{RT}{v}\left[ {\left( {J - 1} \right)\log \left( {1 - \frac{1}{J}} \right) + \chi \left( {1 - \frac{1}{J}} \right)} \right]$$

where the only two parameters are $N$, the number of network chains per unit volume, and $\chi$, the Flory–Huggins interaction parameter. $R$ is the gas constant. Differentiating with respect to $J$, we find:

$$\frac{dW}{{dJ}} = NRT\left( {\frac{1}{{J^{\frac{1}{3}} }} - \frac{1}{J}} \right) + \frac{RT}{v}\left( {\frac{1}{J} + \log \left( {1 - \frac{1}{J}} \right) + \frac{\chi }{{J^{2} }}} \right) = \frac{\mu }{v}$$

In equilibrium in i-PDMS system, the chemical potential will be equal everywhere, and for a pure oil bath the chemical potential of oil is zero, so $\mu = 0$. The equilibrium swelling ratio $J$ can be measured directly, while $N$ can be calculated from the measured shear modulus^30^. For the 10:1 (Polymer:crosslinker) ratio PDMS used in this study, Sotiri et al.^30^ found a number average molecular weight between crosslinks of 3.7 kg mol^−1^, which for a PDMS density of 0.965 kg L^−1^ corresponds to $N = 0.26$ mol L^−1^. For the 10 cSt silicone oil used, they provide a density of $0.935{\text{ kg L}}^{ - 1}$ and a molecular weight of 1250 g mol^−1^, which corresponds to $v = 1.34{\text{ L mol}}^{ - 1}$.

Upon complete polymerization in o-PDMS system, the molar concentration of crosslinker molecules in the diluted system is clearly lower than in the non-diluted system. This lower crosslinking density corresponds to a larger average distance between two crosslinks, leading in turn to a larger average number of monomers $N_{c}$ between crosslinks. Yet, due to the conservation of mass it must be true that $m = \frac{{N_{c} \nu }}{2}$. Thus, it is for $N_{c}$ to be higher in the diluted case if $\nu$ and $m$ are the same. This paradox is resolved if the a true number of effective crosslinks $\nu_{e} < \nu$. The total number of crosslinking molecules $\nu$ can still be incorporated into the network through the creation of loops, in which a crosslinker unites two parts of the same chain rather than two different chains (red arrows in Fig. 7A). These kinds of crosslinks do not contribute to the mechanical network structure, as they are essentially equivalent to a shorter single chain.

As a result, o-PDMS should have significantly lower elastic modulus than i-PDMS of the exact same final composition, in agreement with the observed values (Fig. 1B). We can estimate $N$ of o-PDMS by noting that $G\sim N$. Thus $N_{oPDMS} = N_{iPDMS} G_{oPDMS} /G_{iPDMS}$. Using the measured shear moduli of o-PDMS and i-PDMS (Fig. 1B), we find that $N_{oPDMS} \approx 0.11$ mol L^−1^. These measurements then allow us to solve for $\chi$, and we find for $T = 298 {\text{ K}}$ and the swelling ratio of $J = 1.7$ observed by Sotiri et al.^30^ for free-swelling bulk gels that $\chi \approx 0.61$, which is reasonably close to the value of $\chi = 0.743$ for hexamethyldisiloxane-swollen PDMS, as measured by Favre^31^. The calculated chemical potential of the silicone oil in PDMS as a function of the swelling ratio using these values is shown in Fig. 7B. From this, we can calculate that i-PDMS would need to lose ~ 20% of its initial oil content in order to have a chemical potential equivalent to as-prepared o-PDMS.

#### Liquid overlayer formation in i-PDMS vs. o-PDMS

Previous experiments, such as the shedding of intact biofilms from an i-PDMS coating upon slow removal from water^23^ and the decrease of contact angle hysteresis of water on oil-containing PDMS^32^, strongly suggest the formation of an oil-rich region or liquid overlayer (LOL) on the surface of the PDMS when in contact with water. The formation of such a layer is in line with experimental and theoretical work showing the preferential segregation of oligomers at the surface of an elastic matrix^33^. The driving force for this separation is the preferable interaction of the smaller oligomers with the external environment, which may be energetically driven or driven by the entropic attraction of chain ends to the surface^34^. Wong et al.^32^ used dynamic contact angle hysteresis measurements to estimate the change in interfacial energy due to dynamic surface lubrication of the water–PDMS interface. They found that spontaneous lubrication reduced the interfacial energy by about $11.5\; {\text{mJ}}\;{\text{m}}^{ - 2}$, providing an estimation for the driving force of creating a LOL at the water–PDMS interface.

The free energy cost of removing oil from the PDMS matrix and confining it to an oil-rich region or layer at the interface will be approximately equal to the chemical potential of the oil in the gel, µ. If this cost is comparable to the free energy gain from creating a LOL, then it may inhibit the LOL’s formation. In a saturated i-PDMS gel, $\mu \approx 0\;{\text{J}}/{\text{mol}}$, providing no barrier to LOL formation. However, for o-PDMS $\mu \approx - 100\;{\text{J}}/{\text{mol}}$, which means that the energy cost to remove a 10 nm layer of silicone oil from the o-PDMS matrix is on the order of 1 mJ m^−2^, and 10 mJ m^−2^ for a 100 nm layer. These energy costs are comparable to the estimated driving force for LOL formation, suggesting that LOL formation could be greatly inhibited in o-PDMS compared to i-PDMS.

#### Chemical potential of oil in PDMS under applied stress

If there is an external mechanical stress $\sigma$ on the gel, Cai and Suo^35^ show that the chemical potential of the solvent in the gel can be described by the equations

$$\sigma_{1} = \frac{NRT}{{\lambda_{2} \lambda_{3} }}\left( {\lambda_{1} - \lambda_{1}^{ - 1} } \right) + \frac{RT}{v}[\log \left( {1 - \frac{1}{{\lambda_{1} \lambda_{2} \lambda_{3} }}} \right) + \frac{1}{{\lambda_{1} \lambda_{2} \lambda_{3} }} + \frac{\chi }{{\left( {\lambda_{1} \lambda_{2} \lambda_{3} } \right)^{2} }}] - \frac{\mu }{v}$$

$$\sigma_{2} = \frac{NRT}{{\lambda_{1} \lambda_{3} }}\left( {\lambda_{2} - \lambda_{2}^{ - 1} } \right) + \frac{RT}{v}[\log \left( {1 - \frac{1}{{\lambda_{1} \lambda_{2} \lambda_{3} }}} \right) + \frac{1}{{\lambda_{1} \lambda_{2} \lambda_{3} }} + \frac{\chi }{{\left( {\lambda_{1} \lambda_{2} \lambda_{3} } \right)^{2} }}] - \frac{\mu }{v}$$

$$\sigma_{3} = \frac{NRT}{{\lambda_{1} \lambda_{2} }}\left( {\lambda_{3} - \lambda_{3}^{ - 1} } \right) + \frac{RT}{v}[\log \left( {1 - \frac{1}{{\lambda_{1} \lambda_{2} \lambda_{3} }}} \right) + \frac{1}{{\lambda_{1} \lambda_{2} \lambda_{3} }} + \frac{\chi }{{\left( {\lambda_{1} \lambda_{2} \lambda_{3} } \right)^{2} }}] - \frac{\mu }{v}$$

$$\lambda_{1} \lambda_{2} \lambda_{3} = 1 + vC$$

Using these equations we can solve for the chemical potential of the oil in an o-PDMS gel under uniaxial ($\sigma_{1} = \sigma$, $\sigma_{2} = \sigma_{3} = 0$) and biaxial ($\sigma_{1} = \sigma_{2} = \sigma$, $\sigma_{3} = 0$) stress (Fig. 7C). We see that in either case compression leads to an increase in the chemical potential. We note that experimentally it is not difficult to realize biaxial compression of a coating. It can simply be adhered to a prestretched surface that is relaxed after curing, as is done for dielectric elastomer actuators^36^.

## Discussion

From the results presented we observe that the pre-cure (one-pot) addition of a compatible free silicone oil to a PDMS matrix (o-PDMS treatment) notably improves the fouling-prevention performance of the silicone elastomer. The o-PDMS treatment showed a marked improvement over the oil-free PDMS control in most of the field and lab experiments. The magnitude of the performance improvement achieved with the o-PDMS treatment is well aligned with the previous studies that have investigated the addition of silicone oils to silicone elastomers^17,18,37^. However, the post-cure infusion approach (i-PDMS) shows a much stronger performance that exceeds the performance of the o-PDMS treatment and Intersleek 700, an optimized and commercially relevant one-pot silicone foul-release (FR) treatment for marine applications. This is remarkable, as o-PDMS and i-PDMS tested in this study are essentially identical with regard to their composition. It is important to note that while the composition of both coatings is matching, their materials properties, such as stiffness or ability to form a lubricant overlayer (LOL), are not (Fig. 1).

It can hence be concluded that while both the oil incorporation approach (o-PDMS treatment) and the post-cure infusion approach (i-PDMS treatment) improve upon the fouling prevention/release performance of silicone elastomers, the post-cure infusion approach leads to coatings with distinctly different properties and abilities to combat fouling. An explanation for this unique performance was provided by our mechanistic model describing o-PDMS and i-PDMS based on the Flory–Rehner theory of swelling an elastomer network in a small molecule solvent^29^. Our model suggests that for the two polymerization conditions, both with the same number of polymerizable monomers $m$ and crosslinking molecules $\nu$, o-PDMS must necessarily have a smaller value of $N$ due to the lower density of effective crosslinks, which leads to longer chains and thus fewer chains per volume. As a result, o-PDMS will have significantly lower elastic modulus than i-PDMS, due to the linear relationship between shear modulus and crosslinking density^30^. This prediction matches well with our observed shear moduli of 555 kPa and 1342 kPa for o-PDMS and i-PDMS, respectively (Fig. 1B). The lower shear modulus of o-PDMS may explain some of its enhanced antifouling performance and reduced adhesion strength of fouling organisms compared to neat, oil-free PDMS (Figs. 2, 3, 4, 5, 6), as it has been shown that the shear stress $\tau$ needed to de-adhere a foulant from a soft elastic surface scales as $\tau \sim \left( {W_{adh} E} \right)^{1/2}$ where $E$ is the Young’s modulus and $W_{adh}$ is the work of adhesion^29^. However, the improved performance of i-PDMS, which is 2.4 times stiffer than o-PDMS (Fig. 1B), cannot be explained with this logic.

While some of the barnacle adhesion strength difference between i-PDMS and o-PDMS (Figs. 1D, 6, 7) could also be due to the swelling-induced thickness differences (~ 100 µm for the o-PDMS coating vs. ~ 150 µm for the i-PDMS coating)^38^, with thicker coating requiring less force to detach adhered barnacles, the difference in thickness is too small to explain the magnitude of the differences in de-adhesion forces^31^.

Instead, the improved performance of i-PDMS is likely explained by the formation of a thin stable lubricant overlayer (LOL) on its surface^24^, while both our experimental (Fig. 1C) and theoretical analyses show that a LOL does not form on o-PDMS. This LOL could help to mitigate fouling in multiple ways. First, the lubricant could mask the surface, preventing fouling organisms from recognizing it as a suitable solid substrate^24^. The lubricant overlayer also increases surface slipperiness, minimizing the force / weight required to release attached fouling organisms. Additionally, the LOL is likely responsible for the strong biofilm retraction forces seen in the *C. lytica* bacterial assay (Fig. 2A) and the Scituate field study (Fig. 3B). This strong retraction force could disrupt early fouling community formation with the potential of delaying the fouling process by several months, as seen in Scituate (Fig. 3A) and Morro Bay (Fig. 4) field immersion studies. Additionally, the removal and retraction of the biofilm could reduce settlement cues that would otherwise entice the larval stages of marine fouling organisms to adhere to the coatings^39^. The retraction effects observed in the field only occurred during the air–water interface transition of the treatments during the field surveys and may have little relevance to permanently submerged surfaces. Nevertheless, strong retraction effects are considered to be a good proxy for the adhesion-prevention performance of FR surfaces^40^. In cases where an organism is able to bypass the LOL and settle onto the PDMS surface, such as barnacle adhesion (Fig. 2C,D), the LOL can significantly lower the work of adhesion by leading to the formation of a lower-energy PDMS-oil interface upon de-adhesion, rather than the higher-energy PDMS-water interface that is created on surfaces without a LOL^24^. This is in line with a previous study^41^ that has shown strong repellent properties of slippery surfaces even in a partially de-wetted state.

This raises the question of why o-PDMS coatings do not form a LOL despite having the same oil content as i-PDMS. The answer is again provided by the Flory–Rehner theory of elastomer swelling, in which the free energy decrease due to infiltration of solvent into the elastomer matrix is counteracted by the free energy cost of stretching the polymer chains. Thus, in o-PDMS, where the elastomer is polymerized in a stress-free state, more oil can be incorporated after polymerization before reaching saturation, while the infusion process leads to complete saturation of i-PDMS. The corresponding energy cost to remove oil from the PDMS matrix, the chemical potential µ, is thus essentially zero in i-PDMS, leading to the facile formation of a LOL, while in o-PDMS µ is high enough to maintain the oil in the bulk of the polymer and inhibit its travel to the interface to form a LOL (Fig. 7C). The long-term performance of i-PDMS is, therefore, likely determined by its ability to form and retain a LOL. Our thermodynamic estimations provided above suggest that i-PDMS would need to lose about 20% of its oil loading to have a µ value comparable to o-PDMS. While fouling release events^23^ and shear stresses may remove some lubricant from the surface, this lubricant is quickly replenished and total losses do not approach this 20% threshold. A strong indication that this is the case is the extended longevity that i-PDMS has shown during the Scituate Harbor, Morro Bay and Singapore Harbor field studies (Figs. 3,4,5) , maintaining full performance over the entire study period of six, nine and 24 months, respectively. This i-PDMS performance duration is remarkable for a simple experimental coating procedure, especially as i-PDMS matched and exceeded the performance of the commercial FR coating Intersleek 700 during this period.

The identification of a critical µ value for the formation of a LOL is necessary to assess the longevity of the i-PDMS holistically and to guide the optimal design of FR coatings (FRC). The Flory–Rehner theory indicates that the µ of o-PDMS could potentially be reduced to zero through the application of a compressive stress during polymerization, suggesting a facile laboratory method for conducting these experiments and a potential way to create controllable, on-demand FRCs using the simpler o-PDMS process. This might allow for the application of o-PDMS as slippery coatings with tunable wettability, as previously demonstrated for porous Teflon membranes^42^. Furthermore, the extended fouling prevention performance of i-PDMS has so far been tested in static fouling conditions, which is a critically important aspect as most ship hull fouling occurs when vessels are static^43^. However, the performance, longevity and lubricant retention of i-PDMS under dynamic fouling conditions, representative of a boat or ship moving in water remains an area of continued research interest.

We conclude that the enhanced performance of i-PDMS compared to o-PDMS of the same composition is likely explained by the materials’ different abilities to form and retain a lubricant overlayer, which we explain using the Flory–Rehner theory of the thermodynamics of elastomer swelling. This theory provides guidance for the future design and optimization of FRCs, such as the potential for o-PDMS to form a LOL under biaxial stress, or the optimization of FRC’s composition using fully-biodegradable oils^7^ or unique polymer-oil formulations for which the chemical potential is sufficiently low, indicating the low energy cost of removing oil from the matrix and its travel to the free interface to form LOL. The ease of adding and removing silicone oil from i-PDMS also makes it a highly versatile material for medical applications^44^, such as the harvesting of cell sheets^45^ or the fouling prevention in catheter tubes^46^ and scalpel blades^47^.

Overall, the substantial fouling release performance improvements conveyed by simple post-cure infusion, as shown in this study, may lead to a renewed interest in how silicone oils interact with the silicone matrix and may inform the design of the next generation of fouling-release coatings.

## Methods

A full description of the methods used can be found in the Supplementary Information S1 provided.

## Supplementary Information

Supplementary Information.

## Data Availability

Data is available on request from the authors. Contact Stefan Kolle for further information.
