# Supplementary material for: On the mechanism of marine fouling-prevention performance of oil-containing silicone elastomers
Source: Sci Rep. 2022 Jul 12;12:11799. doi: 10.1038/s41598-022-15553-4 (PMC9276722; doi:10.1038/s41598-022-15553-4)
Supplement: Supplementary file 1 — Supplementary Information. [file 41598_2022_15553_MOESM1_ESM.docx]

**On the mechanism of marine fouling-prevention performance of oil-containing silicone elastomers**

Stefan Kolle^1^, Onyemaechi Ahanotu^2^, Amos Meeks^1^, Shane Stafslien^3^, Michael Kreder^1^, Lyndsi Vanderwal^3^, Lucas Cohen^4^, Grant Waltz^5^, Chin Sing Lim^6^, Dave Slocum^7^, Elisa Maldonado Greene^4^, Kelli Hunsucker^8^, Geoffrey Swain^8^, Dean Wendt^5^, Serena Lay-Ming Teo^6^ and Joanna Aizenberg^1,2^

^1^John A. Paulson School of Engineering and Applied Sciences, Harvard University, Cambridge, Massachusetts 02138, USA.

^2^Wyss Institute for Biologically Inspired Engineering, Harvard University, Cambridge, MA, 02138.

^3^Department of Coatings and Polymeric Materials, North Dakota State University, Fargo, ND 58102.

^4^University of San Diego, 5998 Alcala Park, San Diego, CA 92110

^5^Center for Coastal Marine Sciences, California Polytechnic State University, San Luis Obispo, CA 93407.

^6^Tropical Marine Science Institute, National University of Singapore, Singapore 119227.

^7^Stellwagen Bank National Marine Sanctuary, National Oceanic and Atmospheric Administration, Scituate, MA 02066.

^8^Center for Corrosion and Biofouling Control, Florida Institute of Technology, Melbourne, FL 32901.

Corresponding author:

jaiz@seas.harvard.edu, skolle@seas.harvard.edu

Supplementary Information

**S1. Methods:**

**S1.1. Materials preparation**

***Preparation of PDMS coating:*** Sylgard 184 elastomer kit (Dow Corning Corporation, Midland, MI) was used in the production of the tested silicone coatings. The base (part A) and curing agent (part B) were combined in a 10:1 ratio (A:B) and mixed in a planetary centrifugal mixer (Thinky Corporation, Tokyo, Japan) at 2000 rpm for 1 min, followed by a second mixing step at 2200 rpm for 1 min. After mixing, 10 g of elastomer mixture was applied to the surface of 1.5 cm steel discs (NDSU bacterial biofilm assays), 4” x 8” steel plates (NDSU mussel/barnacle assays and Port Canaveral, Morro Bay and Singapore Harbor field studies), and 1/8” thick, 6^14/16”^x 6^14/16”^ glass plates (Scituate Harbor field study). They were then surface activated using a 2 min, 250W, oxygen plasma (Femto PCCE plasma cleaner, Diener electronic GmbH, Ebhausen, Germany). Excess silicone prepolymer was removed by spinning slides at 1000 rpm for 60 s via spin coating (Spincoat G3P-15, SCS, Indianapolis, USA) to achieve an approximate ~100μm thickness. For the 1.5 cm stainless steel tokens, 3 droplets were applied to the surface from the tip of a spatula and excess prepolymer was removed using a smaller spin-coater setup (Laurell Technologies Corporation, North Wales, PA). After spin-coating the samples were cured in an oven (Binder GmbH, Tuttlingen, Germany) at 70°C for 4 h.

***Preparation of o-PDMS*:** Sylgard 184 elastomer kit was used to prepare one-pot PDMS treatments as described above. Immediately after, Element 14* PDMS 10-A silicone oil (Momentive, Waterford, NY) was added to the 10:1 PDMS prepolymer at a mass loading equal to 50% of the prepolymer mass (the same oil content as infused silicone SLIPS) before mixing using the planetary mixer. For example, 5 g of PDMS oil were added to 10 grams of uncured 10:1 PDMS. After mixing, 10 g of elastomer mixture was applied to the surface of 1.5 cm diameter steel discs (NDSU bacterial biofilm assays), 4” x 8” steel plates (NDSU mussel/barnacle assays and Port Canaveral, Morro Bay and Singapore Harbor field studies), and 1/8” thick, 6^14/16”^x 6^14/16”^ glass plates (Scituate Harbor field study). The surface was then activated using a 2 min, 250W, oxygen plasma. Excess silicone prepolymer was removed by spinning at 300 rpm for 60 s via spin coating. After spin-coating, the samples were cured in an oven at 70°C for 4 h.

***Preparation of i-PDMS*:** Infused silicone slippery coatings were prepared using the PDMS methods specified above, followed by the subsequent infusion of Element 14* PDMS 10-A into the cured PDMS matrix. The cured PDMS surface was first flooded with an overlayer of Element 14* PDMS 10-A and left to infuse at room temperature for 48h to allow the lubricant to fully infiltrate and equilibrate throughout the silicone polymer matrix. Excess lubricant was subsequently removed from the surface before testing by either spin coating samples at 1000 rpm for 60 s, or allowing the excess lubricant to be drained by gravity by tilting the samples at a 90º angle for 24 h.

***Preparation of INTERSLEEK 700*:** Intersleek 700 (International Marine Coatings, Akzo Nobel) was prepared according to manufacturer specifications: Intersleek 757 topcoat was mixed in 15:4:1 (A:B:C) ratios and mixed by hand using a glass stir rod. This mixture was applied via spin coating at 750 rpm for 60 seconds to achieve a coating thickness of ~150μm on the discs, steel plates, and glass plates. The surfaces were then activated using a 2 min oxygen plasma exposure at 250W. Coatings were left to cure for at least 2 days at room temperature before testing.

**S1.2. Materials characterization**

***AFM detection of lubricant layer:*** i-PDMS, o-PDMS and PDMS control surfaces were investigated using atomic force microscope (JPK instruments, CellHesion200) using cantilevers (BudgetSensors) with spring constant of 5.45 N/m. The setpoint was 19.74 nN, the pulling length was 20 µm and the extent speed was 1 µm/s.

**S1.3. Laboratory-based biofouling characterization**

***Laboratory biofouling characterization (North Dakota State University)*:** Prior to biofouling assessments, all coatings prepared on 24-well plates containing 1.5 cm disc samples and 4” x 8” steel plates were immersed for 7 days in a running tap water tank system. Growth assessments in artificial sea water (ASW) extracts of each coating were subsequently carried out to verify that no toxic components were leaching from the materials.

***Bacteria Biofilm Retraction (Cellulophaga lytica)*:** The characterization of bacteria biofilm retraction on coatings prepared in 24-well plates has been described in detail previously ^1,2,3^. Briefly, overnight cultures of the marine bacterium *Cellulophaga lytica* in marine broth were harvested via centrifugation (10,000xg for 10 minutes) and rinsed three times with sterile artificial seawater (ASW). The bacteria were then re-suspended in ASW supplemented with 0.5 g/L of peptone and 0.1 g/L of yeast extract to achieve a final cell density between 10^7^–10^8^ cells/mL. One mL of bacterial suspension was added to each well and incubated at 28 °C for 24 hrs under static conditions to promote cell attachment and biofilm growth. The coatings were subsequently rinsed three times with ASW, allowed to air dry at ambient laboratory conditions for ~1 h, stained with a crystal violet (CV) dye solution (0.3% w/v) for 15 min, rinsed three times with ASW, and air dried. The degree of biofilm retraction on each coating replicate was measured using an automated software tool that calculated the percent surface coverage of CV-stained biofilms from high resolution digital images ^4^ where a low percent surface coverage indicates a high degree of biofilm retraction.

***Microalgae Cell Attachment (Navicula incerta)*:** The characterization of microalgae cell attachment to coatings prepared in 24-well plates has been described in detail previously ^5,6^. Briefly, five-day-old cultures of the microalgae (diatom) *Navicula incerta* were rinsed three times with ASW and re-suspended in Guillard’s F/2 medium to achieve a final cell density of 10^5^ cells.ml^−1^. One ml of the microalgae suspension was added to each well of the coated plates and incubated statically at 18°C for 2 h in an illuminated growth chamber (photon flux density 46 μm m^−2^ s^−1^). The microalgae suspension was subsequently discarded, the coatings were extracted with 1.0 ml of dimethyl sulfoxide for 15 min, and the resulting eluates were transferred to 96-well plates and measured for fluorescence of chlorophyll (Ex: 360 nm; Em: 670 nm) using a Tecan Safire 2 multi-well plate spectrophotometer.

***Barnacle Reattachment and Adhesion (Amphibalanus amphitrite)*:** The laboratory assessment of adult barnacle reattachment and adhesion has been described previously ^1,7,8^. In our tests, 5 adult barnacles (*Amphibalanus amphitrite*) of a testable size (>5mm basal diameter) were dislodged from glass panels coated with Silastic T2 and placed on the coated 4”x8” steel plates. Immobilization templates were then applied to each panel to anchor barnacles to the coated surfaces ^9^ and then transferred to an artificial saltwater aquarium tank system. The reattached barnacles were fed daily with freshly hatched brine shrimp nauplii (*Artemia sp.*). After 14 days of reattachment in the aquarium system, the steel plates were removed and the reattached barnacles were removed in shear mounted to a semi-automated push-off device to measure the peak force at release. The area of the barnacle base plates were measured using a Sigma Scan Pro software package (SigmaScan Pro 5.0, Systat Software, Inc., Richmond, CA). The adhesion strengths were then calculated by normalizing detachment shear force to basal area. Barnacle adhesion for each coating was reported as the mean value of the total number of barnacles that had a measurable detachment force. Barnacles that had no measurable force for detachment were counted as “not attached” and not included in adhesion calculations.

***Marine Mussel Attachment and Adhesion (Geukensia demissa)*:** The assessment of marine mussel attachment and adhesion was carried out using a customized protocol derived from previously published methods ^10,11,12^. Freshly collected adults of the ribbed mussel *Geukensia demissa* (3-5 cm in size) were obtained from the Duke University Marine Laboratory in Beaufort, North Carolina, USA, and housed in an ASW aquarium tank system with continuous monitoring and maintenance of pH (8.0-8.2) and salinity (35 ppt). Prior to attachment studies, a 4 cm section of acetal plastic rod (product# 98873A105, McMaster-Carr) was adhered to the shell of each mussel, perpendicular to the ventral edge, using a 3M acrylic adhesive (product# 7467A135, McMaster-Carr). Custom-designed templates fabricated from PVC sheets were then used to immobilize six mussels onto each coated 4”x8” steel plate, using setscrews to firmly secure the adhered, plastic rods. The samples were placed in the ASW aquarium system and the mussels were fed daily with live marine phytoplankton (DTs Premium Reef Blend Phytoplankton). After three days of immersion, the mussels were removed from the ASW aquarium tank system and the total number of mussels exhibiting attachment of byssus threads was recorded for each surface. The rod of each attached mussel was then secured to an individual 5N load cell of a custom-built, tensile force gauge outfitted with six load cells to enable simultaneous measurements of all attached mussels. The total force required to detach the byssus threads for each mussel was recorded (1 mm/s pull rate) and the average pull-off force value (Newtons) for all attached mussels was calculated for each coating surface.

**S1.4. Field study methods and biofouling characterization**

***Field site (Scituate Harbor, MA, USA)*:** The Scituate Harbor field site (42°11’55” N, 70°43’5” W) is located on the land-ward facing site of a small peninsula with direct access to the Atlantic Ocean. It consists of two floating docks attached to a pier facility owned and maintained by the Stellwagen Bank National Marine Sanctuary (NOAA) that graciously allowed us to use their floating docks for our field experiments. The site was chosen as it receives substantial and regular tidal flushing, while at the same time being protected by a seawall from direct wave impact. The site shows a diverse biological community, typical of the Northeastern Atlantic coast, dominated by mussels (*Mytilus edulis*), tunicates and hydroids during the summer season when the experiments were performed and an assembly of microalgae and filamentous brown macroalgae during the autumn / winter seasons.

***Field site (Morro Bay, CA, USA)*:** The Cal Poly test site is located near the mouth of Morro Bay (35°22'10'' N, 120°51'48” W) and is subjected to a temperate marine environment. It is a floating dock that raises and lowers with the tidal cycle so the panels remain at a constant depth of approximately one meter. The temperature and salinity fluctuate seasonally from 11.2-22.3˚C and 13-35‰. Morro Bay’s fouling community is diverse and changes seasonally. Barnacle recruitment usually occurs from summer to early fall and late winter to spring. The heaviest fouling occurs between spring and fall. The fouling community consists of sponges, tunicates, tubeworms, hydroids, anemones, tube-dwelling amphipods, arborescent and encrusting bryozoans and several species of barnacles, the most abundant of which is *Balanus crenatus*. The most dominant species is an invasive encrusting bryozoan *Watersipora*

*subtorquata*.

***Field site (Port Canaveral, FL, USA)*:** The FIT field site is located inside Port Canaveral (28°24'27'' N, 80°37'38'' W)

along the central east coast of Florida. The port was created in 1953 and is a hub for cruise and cargo ships, US Navy, Coastguard, fishing vessels and recreational boats. The site is located in a subtropical environment and the water temperature fluctuates between 20–32 deg C, with an average salinity of 35 ± 1.2 ppt ^13^. It is an area of high fouling activity with seasonal variation in fouling organisms. The biofouling community in warmer months is dominated by calcareous tubeworms, barnacles, colonial tunicates, and encrusting bryozoans. In cooler months, biofilms and arborescent bryozoans dominate.

***Field site (Singapore Harbor, Singapore)*:** The TMSI test site is located at the Republic of Singapore Yacht Club (RSYC) on the south-west coast of Singapore (1°17’40” N, 103°45’37” E). Surface water temperatures are relatively high for most part of the year, ranging between 27 to 31 °C. Salinities in the near-coastal areas are typically estuarine, and fluctuate between 20-30 ppt. The most common hard macrofouling organisms observed at the site on panels during the period were tubeworms. Several species of serpulid tubeworms may be found at the test site, especially on unprotected surfaces, including *Spirobranchus krausii*, *Hydroides spp*. and *Ficopomatus sp*. Spirorbid worms were typically abundant throughout the year. Three species of barnacles, *Amphibalanus reticulatus*, *A. cirratus* and *A. amphitrite* occur. The most common mollusc occurring on the panels were *Dendrostrea cf. foliaceum* and *Anomia sp*. Soft-fouling on the panels was dominated by encrusting sponges and colonial tunicates. Bryozoans such as *Bugula sp.* occurs sporadically but the actual fouling cover recorded is always low as the area of contact with the panel surfaces is small and they are usually attached to secondary substrata. Slime coverage was aggressive on all substrates especially during the NE monsoon months.

***Field survey methodology and fouling observations (Scituate Harbor)*:** PVC settlement panels were placed through slots of a floating raft, moored to a floating dock, maintaining a constant submersion depth of samples of 0.5 m. Each panel had four retaining frames, coated with anti-fouling copper paint (ACT, Interlux, Akzo Nobel) to reduce the edge effect, used for securing the 175 x 175 mm^2^ glass settlement plates to each of the panels. Field observational surveys were conducted every other week during the entire summer testing season, when the mussel attachment takes place. Surveys were conducted every two weeks. The panels containing test substrates were removed from the water and photographed horizontally, observations in fouling and coating condition were also made while the panel was out of the water. A white balance card (Digital Kolor Kard 5x7, DGK Color Tools, Boston, USA) was included in each photo taken. The in-air survey time of each panel was limited to 10 min, before returning the panels to the water, minimizing the survey impact on community development.

***Image analysis (Scituate Harbor)*:**  Preprocessing of the photos was conducted using a python script, to perform a white balance, vignette removal and 0.5 inch crop of the sample edge on each image. Subsequently the images were loaded into Coral Point Count with Excel extensions (CPCe 4.1) from the National Coral Reef Institute (NCRI) at Nova Southeastern University ^14^. Using CPCe, 50 random points were overlaid on top of the image and the marine fouling organism attached at each point was identified by a user trained in the identification of marine benthic communities. The graphs showing the community composition were plotted using Excel (Microsoft Corporation).

***Mussel settlement counts (Scituate Harbor)*:** The quantification of the mussel settlement was conducted with the photos taken during week 8 field survey, when the mussel spat reached a sufficient size to be visible on the high-resolution images. Each image was divided into 100 squares of equal size (2.32 cm^2^). Five randomly selected squares were taken per image and all mussels found in these squares were manually counted using a Tally Counter. Subsequently an estimate of the mussel spat per cm^2^ was made based on these counts.

***Fouling observations (Morro Bay, Port Canaveral, Singapore Harbor)*:** Digital photographs of each panel replicate were taken on a monthly basis. Percent coverage of fouling organisms were visually estimated in the field for each replicate according to ASTM D6990-05. Panel edges were blocked out to eliminate any potential edge effects so the area of each panel assessed lies within the rectangular area lying within the corner holes. Total fouling coverage and composition was either visually estimated in the field (Morro Bay) or through image analysis using CPCe 4.1 (Port Canaveral) or Photogrid 1.0 (Singapore Harbor) with 50 random sample point estimate method.

***Hard-fouling field adhesion studies (Morro Bay and Port Canaveral)*:** Hard-fouling adhesion studies of barnacles were conducted at Port Canaveral according to D5618-94 by selecting life barnacles, applying shear force to the base of the organism and measuring the removal force. Adhesion failure must be between the organism and the surface for a reading to be valid. The removed organisms are retained and returned to the laboratory where their base plate is measured with a scanneror from measurements of the basal plate diameter in the field ^15^. The shear strength of adhesion (MPa) is calculated by dividing the force of removal (Newtons) by the area of the organism base (square millimeters). At the Morro Bay field site this methodology has been adapted for the use on encrusting bryozoans ^15^.

***Statistical analysis*:** The statistical analysis and bar charts of the adhesion and count data was conducted with GraphPad Prism 8.0.2. Comparative analysis between the treatments was conducted as unpaired t-tests.

**S2. Statistical Analysis**

**S2.1 Laboratory Evaluation of i-PDMS and o-PDMS fouling prevention performance**

| **Table 1)** Results summary table for the laboratory fouling assays, showing means and standard deviation of each coating tested. H = comparison with average historical adhesion data, due to Intersleek 700 coating failure (poor batch performance). | | | | | | | | |
| --- | --- | --- | --- | --- | --- | --- | --- | --- |
| **Treatment** | ***C.lytica* assay** | | ***N.incerta* assay** | | ***G.demissa* assay** | | ***A.amphitrite* assay** | |
|  | **N** | **coverage in**  **%** | **N** | **fluorescence in RFU** | **N** | **Adhesion in Newton** | **N** | **Adhesion in**  **MPa** |
| **PDMS control** | 3 | 99.65 ± 0.53 | 3 | 5956 ± 540 | 6 | 16.63 ± 7.05 | 3 | 0.188 ± 0.065 |
| **Intersleek 700** | 3 | 34.74 ± 22.83 | 3 | 4811 ± 75 | 5 | 7.54 ± 4.88 | 176 | 0.074 ± 0.033 (H) |
| **o-PDMS** | 3 | 99.03 ± 0.83 | 3 | 5891 ± 663 | 5 | 8.54 ± 3.58 | 5 | 0.064 ± 0.060 |
| **i-PDMS** | 3 | 7.41 ± 5.74 | 3 | 6061 ± 561 | 5 | 0 | 3 | 0.018 ± 0.003 |

| **Table 2)** unpaired t-test results for *C.lytica* | | | | |
| --- | --- | --- | --- | --- |
| **Test details** | **P-value** | **Sig.** | **t** | **df** |
| PDMS vs. IS700 | 0.0079 | ****** | 4.932 | 4 |
| PDMS vs. o-PDMS | 0.3351 | **no** | 1.095 | 4 |
| PDMS vs. i-PDMS | <0.0001 | ******** | 27.72 | 4 |
| IS700 vs. o-PDMS | 0.0082 | ****** | 4.874 | 4 |
| IS700 vs. i-PDMS | 0.1147 | **no** | 2.011 | 4 |
| o-PDMS vs. i-PDMS | <0.0001 | ******** | 27.37 | 4 |

| **Table 3)** unpaired t-test results for *N.incerta* | | | | |
| --- | --- | --- | --- | --- |
| **Test details** | **P-value** | **Sig.** | **t** | **df** |
| PDMS vs. IS700 | 0.022 | ***** | 3.638 | 4 |
| PDMS vs. o-PDMS | 0.9026 | **no** | 0.1304 | 4 |
| PDMS vs. i-PDMS | 0.8257 | **no** | 0.2350 | 4 |
| IS700 vs. o-PDMS | 0.0486 | ***** | 2.804 | 4 |
| IS700 vs. i-PDMS | 0.0187 | ***** | 3.822 | 4 |
| o-PDMS vs. i-PDMS | 0.7517 | **no** | 0.3389 | 4 |

| **Table 4)** unpaired t-test results for *G.demissa* | | | | |
| --- | --- | --- | --- | --- |
| **Test details** | **P-value** | **Sig.** | **t** | **df** |
| PDMS vs. IS700 | 0.0379 | ***** | 2.431 | 9 |
| PDMS vs. o-PDMS | 0.0458 | ***** | 2.315 | 9 |
| PDMS vs. i-PDMS | 0.0005 | ******* | 5.230 | 9 |
| IS700 vs. o-PDMS | 0.7203 | **no** | 0.3710 | 8 |
| IS700 vs. i-PDMS | 0.0086 | ****** | 3.457 | 8 |
| o-PDMS vs. i-PDMS | 0.0007 | ******* | 5.333 | 8 |

| **Table 5)** unpaired t-test results for *A.amphitrite* | | | | |
| --- | --- | --- | --- | --- |
| **Test details** | **P-value** | **Sig.** | **t** | **df** |
| PDMS vs. IS700 | <0.0001 | ******** | 5.825 | 177 |
| PDMS vs. o-PDMS | 0.034 | ***** | 2.734 | 6 |
| PDMS vs. i-PDMS | 0.0105 | ***** | 4.545 | 4 |
| IS700 vs. o-PDMS | 0.5325 | **no** | 0.6254 | 179 |
| IS700 vs. i-PDMS | 0.0036 | ****** | 2.948 | 177 |
| o-PDMS vs. i-PDMS | 0.2409 | **no** | 1.301 | 6 |

**S2.2 Mussel spat density analysis, Scituate Harbor, MA, week 8**

| **Table 6)** Mussel spat densities in Scituate Harbor, MA | | |
| --- | --- | --- |
| **Treatment** | **N** | **Mussel spat densities per cm^2^** |
| **PDMS control** | 5 | 99.12 ± 42.76 |
| **Intersleek 700** | 5 | 14.28 ± 7.35 |
| **o-PDMS** | 5 | 39.34 ± 23.15 |
| **i-PDMS** | 5 | 3.4 ± 2.58 |

| **Table 7)** unpaired t-test results for Mussel spat densities | | | | |
| --- | --- | --- | --- | --- |
| **Test details** | **P-value** | **Sig.** | **t** | **df** |
| PDMS vs. IS700 | 0.0024 | ****** | 4.372 | 8 |
| PDMS vs. o-PDMS | 0.0251 | ***** | 2.749 | 8 |
| PDMS vs. i-PDMS | 0.0011 | ****** | 4.996 | 8 |
| IS700 vs. o-PDMS | 0.0499 | ***** | 2.307 | 8 |
| IS700 vs. i-PDMS | 0.0142 | ***** | 3.122 | 8 |
| o-PDMS vs. i-PDMS | 0.0087 | ****** | 3.451 | 8 |

| **Table 8)** Summary table of the encrusting bryozoan and barnacle adhesion studies in Morro Bay, showing means and standard deviations of all tested coatings. | | | | |
| --- | --- | --- | --- | --- |
| **Treatment** | **Encrusting bryozoan adhesion** | | **Barnacle adhesion** | |
|  | **N** | **(in N/mm^2^)** | **N** | **(in N/mm^2^)** |
| **PDMS control** | 3 | 0.165 ± 0.012 | 14 | 0.299 ± 0.023 |
| **Intersleek 700** | 8 | 0.077 ± 0.009 | 8 | 0.109 ± 0.015 |
| **o-PDMS** | 3 | 0.094 ± 0.017 | 11 | 0.186 ± 0.015 |
| **i-PDMS** | 3 | 0.03 ± 0.001 | 0 | 0 (no barnacles) |

**S2.3 Encrusting bryozoan and barnacle adhesion in Morro Bay, CA**

| **Table 9)** unpaired t-test results for encrusted bryozoan adhesion analysis | | | | |
| --- | --- | --- | --- | --- |
| **Test details** | **P-value** | **Sig.** | **t** | **df** |
| PDMS vs. IS700 | <0.0001 | ******** | 13.34 | 9 |
| PDMS vs. o-PDMS | 0.0041 | ****** | 5.91 | 4 |
| PDMS vs. i-PDMS | <0.0001 | ******** | 19.42 | 4 |
| IS700 vs. o-PDMS | 0.053 | **no** | 2.226 | 9 |
| IS700 vs. i-PDMS | <0.0001 | ******** | 8.731 | 9 |
| o-PDMS vs. i-PDMS | 0.0029 | ****** | 6.509 | 4 |

| **Table 10)** unpaired t-test results for encrusted bryozoan adhesion analysis | | | | |
| --- | --- | --- | --- | --- |
| **Test details** | **P-value** | **Sig.** | **t** | **df** |
| PDMS vs. IS700 | <0.0001 | ******** | 20.85 | 20 |
| PDMS vs. o-PDMS | <0.0001 | ******** | 14.08 | 23 |
| PDMS vs. i-PDMS | <0.0001 | ******** | 21.95 | 15 |
| IS700 vs. o-PDMS | <0.0001 | ******** | 11.05 | 17 |
| IS700 vs. i-PDMS | <0.0001 | ******** | 12.17 | 9 |
| o-PDMS vs. i-PDMS | <0.0001 | ******** | 20.85 | 12 |

**S2.4 Barnacle adhesion in Port Canaveral, FL**

| **Table 11)** Summary table of the barnacle adhesion studies in Port Canaveral, showing means and standard deviations of all tested coatings. | | | | |
| --- | --- | --- | --- | --- |
| **Treatment** | **Barnacle adhesion (month 4)** | | **Barnacle adhesion (month 7)** | |
|  | **N** | **MPa** | **N** | **MPa** |
| **PDMS control** | 12 | 0.24 ± 0.09 | 6 | 0.16 ± 0.05 |
| **Intersleek 700** | 7 | 0.06 ± 0.03 | 3 | 0.08 ± 0.04 |
| **o-PDMS** | 14 | 0.14 ± 0.06 | 6 | 0.11 ± 0.05 |
| **i-PDMS** | 9 | 0.04 ± 0.02 | 5 | 0.04 ± 0.01 |

| **Table 12)** unpaired t-test results barnacle adhesion (month 4) | | | | |
| --- | --- | --- | --- | --- |
| **Test details** | **P-value** | **Sig.** | **t** | **df** |
| PDMS vs. IS700 | <0.0001 | ******** | 5.076 | 17 |
| PDMS vs. o-PDMS | 0.0025 | ****** | 3.378 | 24 |
| PDMS vs. i-PDMS | <0.0001 | ******** | 6.507 | 19 |
| IS700 vs. o-PDMS | <0.0038 | ****** | 3.297 | 19 |
| IS700 vs. i-PDMS | 0.06 | **no** | 1.601 | 14 |
| o-PDMS vs. i-PDMS | <0.0001 | ******** | 4.797 | 21 |

| **Table 13)** unpaired t-test results barnacle adhesion (month 7) | | | | |
| --- | --- | --- | --- | --- |
| **Test details** | **P-value** | **Sig.** | **t** | **df** |
| PDMS vs. IS700 | 0.0482 | ***** | 2.389 | 7 |
| PDMS vs. o-PDMS | 0.1139 | **no** | 1.732 | 10 |
| PDMS vs. i-PDMS | 0.0005 | ******* | 5.234 | 9 |
| IS700 vs. o-PDMS | 0.4 | **no** | 0.896 | 7 |
| IS700 vs. i-PDMS | 0.08 | **no** | 2.236 | 6 |
| o-PDMS vs. i-PDMS | 0.0137 | ***** | 3.053 | 9 |

**References:**

1. Stafslien, S. J., Bahr, J., Daniels, J., Christianson, D. A., & Chisholm, B. J. (2011). High-Throughput Screening of Fouling-Release Properties: An Overview. *J Adhes Sci Technol*, **25**(17), 2239-2253. doi:10.1163/016942411X574934
2. Stafslien, S., Daniels, J., Chisholm, B., & Christianson, D. (2007). Combinatorial materials research applied to the development of new surface coatings III. Utilisation of a high-throughput multiwell plate screening method to rapidly assess bacterial biofilm retention on antifouling surfaces. *Biofouling*, **23**(1), 37-44. doi:10.1080/08927010601127311
3. Stafslien, S. J., et al. (2007). Combinatorial materials research applied to the development of new surface coatings VI: An automated spinning water jet apparatus for the high-throughput characterization of fouling-release marine coatings. *Rev. Sci. Instrum*., **78**(7), 072204. doi:10.1063/1.2755965
4. Ribeiro, E., et al. (2008). Automated Image-Based Method for Laboratory Screening of Coating Libraries for Adhesion of Algae and Bacterial Biofilms. *J. Comb. Chem.*, **10**(4), 586-594. doi:10.1021/cc800047s
5. Casse, F., et al. (2007). Combinatorial materials research applied to the development of new surface coatings V. Application of a spinning water-jet for the semi-high throughput assessment of the attachment strength of marine fouling algae. Biofouling, **23**(2), 121-130. doi:https://doi.org/10.1080/08927010701189583
6. Sokolova, A., et al. (2012). A comparison of the antifouling/foul-release characteristics of non-biocidal xerogel and commercial coatings toward micro- and macrofouling organisms. *Biofouling*, **28**(5), 511-523.

doi:10.1080/08927014.2012.690197

1. Rittschof, D., et al. (2008). Barnacle reattachment: a tool for studying barnacle adhesion. *Biofouling*, **24**(1), 1-9. doi:10.1080/08927010701784920
2. Stafslien, S. J., et al. (2016). Comparison of laboratory and field testing performance evaluations of siloxane-polyurethane fouling-release marine coatings. *Biofouling*, **32**(8), 949-968. doi:10.1080/08927014.2016.1211269
3. Stafslien, S., et al. (2012). An improved laboratory reattachment method for the rapid assessment of adult barnacle adhesion strength to fouling-release marine coatings. *J Coat Technol Res*, **9**(6), 651-665. doi:10.1007/s11998-012-9409-7
4. Bell, E. C., & Gosline, J. M. (1997). Strategies for life in flow:tenacity, morphometry, and probability of dislodgment of two Mytilus species. *Mar. Ecol. Prog. Ser.*, **159**, 197-208. doi:10.3354/meps159197
5. Burkett, J. R., Wojtas, J. L., Cloud, J. L., & Wilker, J. J. (2009). A Method for Measuring the Adhesion Strength of Marine Mussels. *J Adhes*, **85**(9), 601-615. doi:10.1080/00218460902996903
6. Crisp, D. J., Walker, G., Young, G. A., & Yule, A. B. (1985). Adhesion and substrate choice in mussels and barnacles. *J. Colloid Interface Sci.*, **104**(1), 40-50. doi:https://doi.org/10.1016/0021-9797(85)90007-4
7. Hunsucker, K.Z., et al. 2019. Using ultraviolet light for improved antifouling performance on ship hull coatings. *Biofouling*, **35**(6), pp.658-668.
8. Kohler, K. E., & Gill, S. M. (2006). Coral Point Count with Excel extensions (CPCe): A Visual Basic program for the determination of coral and substrate coverage using random point count methodology. *Comput. Geosci*, **32**(9), 1259-1269. doi:https://doi.org/10.1016/j.cageo.2005.11.009
9. Waltz, G. T., Hunsucker, K. Z., Swain, G., & Wendt, D. E. (2020). Using encrusting bryozoan adhesion to evaluate the efficacy of fouling-release marine coatings. *Biofouling*, **36**(10), 1149-1158. doi:10.1080/08927014.2020.1857742

***Statistical analysis*:** The statistical analyses and bar charts of the adhesion and count data were conducted with GraphPad Prism 8.0.2. Comparative analysis between the treatments was conducted as unpaired t-tests.
